# Triply Enhanced Immunotherapy via Dual Glycan Reforming Integrated with Perforation

**DOI:** 10.1002/advs.202304971

**Published:** 2023-10-23

**Authors:** Yuanjiao Yang, Yuru Wang, Zhicong Chao, Yuhui Yang, Yanyun Fang, Ying Liu, Lin Ding, Yunlong Chen, Huangxian Ju

**Affiliations:** ^1^ State Key Laboratory of Analytical Chemistry for Life Science, School of Chemistry and Chemical Engineering Nanjing University Nanjing 210023 China

**Keywords:** glycan reforming, immunotherapy, perforation, sialic acid, tumor

## Abstract

The enhancement of immunotherapy is an emerging direction to develop highly effective and practical cancer therapeutic methods. Here a triply enhanced immunotherapy drug (TEID) is designed for ingeniously integrating in situ dual glycan reforming with perforation on cell membrane. The TEID is composed of galactose and neuraminidase conjugated streptolysin O (SLO‐Gal and SLO‐NEU), which are encapsulated in a hyaluronic acid (HA) shell for targeted recognition to tumor tissue via cell surface CD44. After targeted delivery and HAase‐mediated degradation in the tumor region, the TEID releases SLO‐Gal and SLO‐NEU, which can easily anchor Gal and NEU on the tumor cell membrane via the perforation of SLO to perform dual glycan reforming for the introduction of Gal and the cleavage of sialic acid. The former can activate immune cells to secret cytokines for immune‐killing, and the latter can weaken the immune inhibition to improve the immunotherapeutic efficacy. Meanwhile, the perforation of SLO can promote the delivery of cytokines into the tumor cells to further enhance the efficacy. The designed triply enhanced immunotherapy strategy opens a significant and promising route to promote clinical immunotherapy of cancer.

## Graphical abstract

A triply enhanced immunotherapy strategy is achieved by integrating the introduction of galactose and the cleavage of sialic acid with perforation on the tumor cell membrane, which simultaneously promotes immune activation, weakens immune inhibition, and stimulates cytokines secretion for effective immunotherapy of cancer.

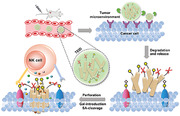

## 1 Introduction

Immunotherapy is one of the most revolutionary therapeutic methods for cancer, which leverages the natural immune system to defeat tumor cells and protect healthy cells.^[^ ^1^ ^]^ The enhancement of immunotherapy has become an emerging direction to develop highly effective and practical cancer therapeutic methods. Generally, the efficacy of immunotherapy is determined by three key aspects, the weakened resistance of tumor cells, the enhanced activation of immune cells, and the promotion of immune‐killing.^[^ ^2^ ^]^ Many crafty tumor cells have evolved various ways to resist the immune system for survival, which mainly utilize the overexpression of the inhibitory receptor‐binding ligands to block the recognition between immune cells and tumor cells.^[^ ^3^ ^]^ The glycosylation of these ligands, especially the sialylation, often plays critical functions in many tumor cell progressions,^[^ ^4^ ^]^ including decreased immunogenicity and increased tumor immune evasion.^[^ ^2^, ^5^ ^]^ On the other hand, the activation of immune cells can be enhanced by many signal response pathways, including the blockade of immune checkpoint^[^ ^6^ ^]^ and the binding between cells‐activating receptors and ligands.^[^ ^2^, ^4^, ^7^ ^]^ Among these immune cells, natural killer (NK) cells are a type of lymphocyte and play critical roles in the innate immunity against tumors.^[^ ^5^, ^8^ ^]^ Especially, the activation of NK cells can be enhanced by specific glycans,^[^ ^9^ ^]^ which are widely present on mammalian cell surfaces. In addition, the immune‐killing can be initiated by releasing the cytotoxic granules including pore‐forming perforin proteins and serine proteases‐granzymes (especially granzyme B, GrB) to lyse tumor cells.^[^ ^10^ ^]^ The perforation is a decisive and membrane‐disruptive step to form pores on tumor cells^[^ ^11^ ^]^ and assist GrB for inducing cell death.^[^ ^10^, ^12^ ^]^

At present, the enhancement of immunotherapy by separately blocking the immune inhibition or activating immune cells is steadily popular. The blockage between the sialic acid (SA) on tumor cells and the sialic acid‐binding immunoglobulin‐like lectins (Siglecs) on immune cells is the most common method, which can transmit immunosuppressive signals to relieve immune inhibition and promote immune enhancement.^[^ ^2^, ^5^ ^]^ This blockage can be achieved by the inhibition of intracellular sialyltransferase,^[^ ^13^ ^]^ or direct cleavage by the sialidase anchored on the tumor cell surface.^[^ ^2^, ^14^ ^]^ The activation of immune cells is usually applied to promote the targeting and the secretion of cytokines from NK cells by increasing the NK‐activating glycan on tumor cells.^[^ ^10^, ^15^ ^]^ In particular, the galactose (Gal) or fructose‐terminated glycoconjugates have been reported to have strong activating abilities for NK cells.^[^ ^9^, ^15^ ^]^ Despite of the certain success accomplished by these methods, the efficiency of single enhancement is always limited. Besides, the enhancement of the perforating process in the immune‐killing has not yet received attention.

**Figure 1:**
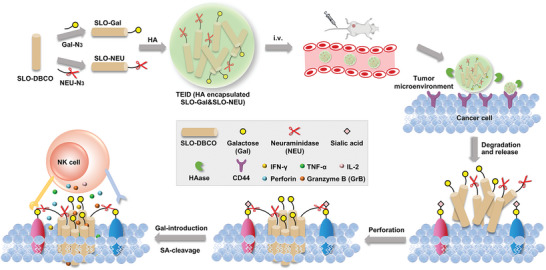
Schematic illustration of triply enhanced immunotherapy via integrating dual glycan reforming and perforation. SLO‐Gal and SLO‐NEU prepared by conjugating Gal and NEU with SLO‐DBCO are encapsulated in HA to obtain TEID for injection into tumor‐bearing mice. After delivered to tumor region by targeting cell surface CD44, TEID is degraded by HAase in the tumor microenvironment to release SLO‐Gal and SLO‐NEU for performing perforation, Gal‐introducing, and SA‐cleaving functions, which triply enhances NK cell‐based immunotherapy.

To break through the limited immunotherapeutic efficacy, this work ingeniously integrates the weakening of immune cell inhibition and the boosting of immune cell activation with extra perforation assistance to achieve for the first time triple enhancement of the immunotherapy. The endogenous perforin is difficult to be utilized in immunotherapy due to its susceptibility to environment or modification.^[^ ^12a^ ^]^ To introduce the perforation assistance, streptolysin O (SLO), a bacterium exogenously‐secreted pore‐forming toxin, is used to construct the triply enhanced immunotherapy drug (TEID). SLO possesses excellent perforating activity and stability after different modifications.^[^ ^16^ ^]^ Upon decoration with dibenzocyclooctyne‐sulfo‐N‐hydroxysuccinimidyl (DBCO‐sulfo‐NHS) ester, the formed SLO‐DBCO can be conveniently connected with azide functionalized galactose (Gal‐N_3_) or α2‐3,6,8 neuraminidase A (NEU‐N_3_) through copper‐free click chemistry^[^ ^17^ ^]^ to obtain SLO‐Gal and SLO‐NEU (**Figure** **1**). After co‐encapsulating SLO‐Gal and SLO‐NEU in hyaluronic acid (HA) cross‐linked shell, the constructed TEID can be specifically recognized by a cluster of differentiation 44 (CD44) on the tumor cell surface to achieve targeted delivery and HAase‐mediated degradation in the tumor microenvironment,^[^ ^18^ ^]^ which releases SLO‐Gal and SLO‐NEU to anchor the NEU and Gal on cell membrane via perforation. The anchored NEU can cleave the original cell surface SA to weaken immune cell inhibition, while the anchored Gal increases the amount of cell surface Gal and thus boosts NK cell activation. Furthermore, the perforation promotes the delivery of secreted GrB to greatly accelerate the immune‐killing process. As a proof of concept, 4T1 tumor‐bearing mice are used as models to demonstrate the triple immunotherapeutic enhancements of the designed TEID. The integration of in situ dual glycan reforming with perforation provides a powerful strategy to improve the clinical immunotherapeutic efficacy of cancer.

## 2 Results and Discussion

### 2.1 Characterization of SLO‐Gal and SLO‐NEU

Gal‐N_3_ obtained by deacetylation of 4AC‐Gal‐N_3_^[^ ^19^ ^]^ showed an *m/z* value of 360, obviously lower than 528 of 4AC‐Gal‐N_3_ (Figure S1a,b, Supporting Information). The mass spectra of SLO and SLO‐DBCO exhibited obvious variation of their m/z values from 60 206 to 61 394 (Figure S1c, Supporting Information), indicating the successful conjugation of two DBCO to a single SLO. Upon copper‐free click linkage^[^ ^17^ ^]^ of SLO‐DBCO with Gal‐N_3_, the obtained SLO‐Gal exhibited an *m/z* value of 62 087, indicating that two Gal were conjugated to SLO due to the presence of two DBCO.

The *m/z* values of NEU and NEU‐N_3_ were 73 482 and 74 621, respectively (Figure S1d, Supporting Information), indicating the presence of two N_3_ on a single NEU. The conjugation of SLO‐DBCO with NEU‐N_3_ was characterized by the cleaving performance of NEU and SLO‐NEU with 2′‐(4‐methylumbelliferyl)‐α‐D‐N‐acetylneuraminic acid (MuNeuNAc) as the substrate. After incubating MuNeuNAc with NEU or SLO‐NEU, both mixtures showed the fluorescence of methylumbelliferone. At an incubation time of 70 min, the fluorescence intensity of SLO‐NEU incubated MuNeuNAc was 80% of NEU incubated MuNeuNAc (Figure S2, Supporting Information), demonstrating the good maintenance of the enzymatic activity of SLO‐NEU.

### 2.2 Functions of SLO‐Gal and SLO‐NEU on Tumor Cells

**Figure 2:**
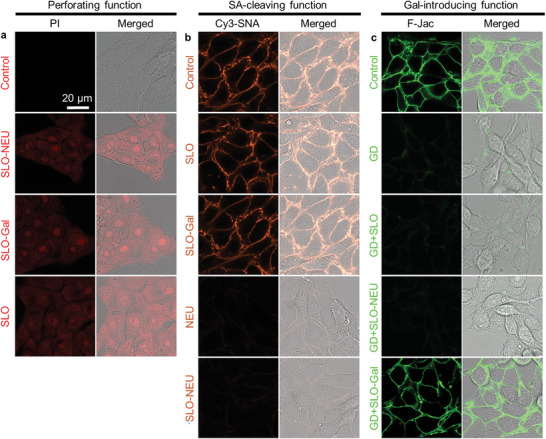
Verification of triple functions of SLO‐Gal and SLO‐NEU on 4T1 cells. a,b) CLSM images of 4T1 cells after incubated with PBS (Control), SLO, SLO‐Gal, or SLO‐NEU and then stained with PI to verify perforating function (a), and with PBS (Control), SLO, SLO‐Gal, NEU or SLO‐NEU and then stained with Cy3‐SNA to verify the SA‐cleaving function (b). c) CLSM images of 4T1 cells and GD per‐treated 4T1 cells after incubated with PBS (Control and GD), SLO, SLO‐Gal, or SLO‐NEU and then stained with F‐Jac to verify Gal‐introducing function.

The functions of SLO‐Gal and SLO‐NEU on tumor cells were first investigated by incubating mouse breast cancer 4T1 cells or human breast cancer MCF‐7 cells with SLO, SLO‐Gal, NEU, or SLO‐NEU, and then staining them with propidium iodide (PI) or Cy3 labeled Sambucus Nigra lectin (Cy3‐SNA), which can specifically recognize SA on the cell surface.^[^ ^20^ ^]^ All the SLO‐NEU, SLO‐Gal, or SLO treated and then PI stained 4T1 (**Figure** **2**a) and MCF‐7 cells (Figure S3a, Supporting Information) showed obvious PI fluorescence, indicating that the SLO‐Gal and SLO‐NEU maintained similar perforating function of SLO on the tumor cell membrane. The CLSM images of SLO and SLO‐Gal treated and then Cy3‐SNA stained 4T1 and MCF‐7 cells exhibited Cy3 fluorescence similar to the Control on cell surface, while the Cy3 fluorescence disappeared on SLO‐NEU or NEU treated cells (Figure 2b; Figure S3b, Supporting Information), demonstrating the SA‐cleaving function of SLO‐NEU on cell membrane.

After NK cells were treated with SLO‐NEU, SLO‐Gal or SLO, and stained with PI, they did not exhibit any PI fluorescence (Figure S4, Supporting Information), indicating that SLO could not perforate on NK cells, consistent to previous reports on the immune‐killing,^[^ ^21^ ^]^ which was beneficial to maintain the viability of NK cells during immunotherapy.

To examine the Gal‐introducing function of SLO‐Gal, the original Gal on the cell membrane was firstl cleaved by incubating the cells with β‐galactosidase (GD). The GD‐treated cells were then incubated with SLO, SLO‐Gal, or SLO‐NEU, and stained with fluorescein labeled Jacalin (F‐Jac), which can specifically recognize Gal on the cell surface.^[^ ^22^ ^]^ Only the GD‐treated 4T1 and MCF‐7 cells incubated with SLO‐Gal showed obvious fluorescence of fluorescein, as the Control cells (Figure 2c; Figure S3c, Supporting Information), indicating a Gal‐introducing process.

The flow cytometric analysis also showed the increasing fluorescence of PI and F‐Jac and the decreasing fluorescence of Cy3‐SNA on 4T1 and MCF‐7 cells upon the similar treatments, further demonstrating the perforating, Gal‐introducing, and SA‐cleaving functions of SLO‐Gal and SLO‐NEU on tumor cells, respectively (Figure S5, Supporting Information). Thus, the SLO‐Gal and SLO‐NEU could successfully perform triple functions on the membranes of different tumor cells.

### 2.3 SLO‐Gal and SLO‐NEU Enhanced in Vitro Immune‐Killing

The in vitro immune‐killing was investigated by incubating 4T1 and MCF‐7 cells with Gal, NEU, SLO, SLO‐Gal, SLO‐NEU, or the mixture of SLO‐Gal and SLO‐NEU (SLO‐Gal&SLO‐NEU), and then with NK cells at a ratio of 1:1 for different times to perform CCK8 assay. With the increasing incubation time, the viability of these incubated 4T1 and MCF‐7 cells obviously decreased (Figure S6, Supporting Information), which indicated the universal immune‐killing ability of NK cells. In addition, the cell viability of the untreated or Gal, NEU, SLO, SLO‐Gal, SLO‐NEU, or SLO‐Gal&SLO‐NEU treated tumor cells exhibited the in‐turn decrease, which demonstrated the enhancing immune‐killing ability, and that SLO‐Gal&SLO‐NEU led to the significantly stronger immune‐killing ability of NK cells than Gal, NEU, SLO, SLO‐Gal, or SLO‐NEU. Thus, the integration of Gal‐introduction, SA‐cleavage, and perforation could exactly triply enhance the immune‐killing of NK cells.

The enhancement of SLO‐Gal&SLO‐NEU on the immune‐killing ability of different effectors (E) was examined with CCK8 assay after incubating SLO‐Gal&SLO‐NEU treated MCF‐7 or 4T1 cells with T cells, peripheral blood mononuclear cells (PBMCs) and NK cells, which demonstrated that SLO‐Gal&SLO‐NEU possessed significantly stronger enhancement on NK cells than on both T cells and PBMCs. Therefore, SLO‐Gal&SLO‐NEU mainly activated NK cells to enhance NK‐induced immune‐killing (Figure S7, Supporting Information).

The ratio of NK cells to tumor cells in the in vitro immune‐killing was optimized by incubating SLO‐Gal&SLO‐NEU treated MCF‐7 or 4T1 cells with NK cells at 10:1, 1:1, 1:10, 1:50, and 1:100 to perform CCK8 assay. Using untreated tumor cells as the control, the enhancing efficiency of immune‐killing was calculated with (ViabilityNK–ViabilitySLO‐Gal&SLO‐NEU+NK)/ViabilityNK. The largest enhancing efficiency was both at the ratio of 1:10 for both 4T1 and MCF‐7 cells (Figure S8, Supporting Information).

**Figure 3:**
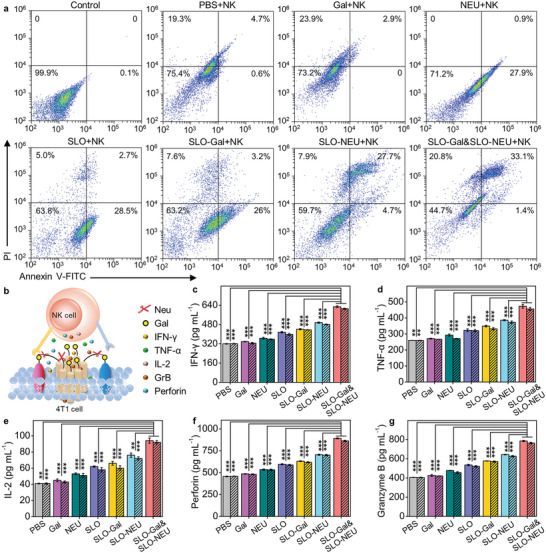
Cytotoxicity and enhanced release of immune‐cytokines. a) Flow cytometric analysis of 4T1 cells after incubated with PBS, Gal, NEU, SLO, SLO‐Gal, SLO‐NEU, or SLO‐Gal&SLO‐NEU and then NK cells, and stained with Annexin V‐FITC and PI. b) Schematic illustration of cytokines (IFN‐γ, TNF‐α, IL‐2, perforin, and GrB) secreted from NK cells in triply enhanced immune‐killing. c–g) ELISA analysis of the secreted cytokines from NK cells after incubation with PBS, Gal, NEU, SLO, SLO‐Gal, SLO‐NEU, or SLO‐Gal&SLO‐NEU pre‐treated 4T1 cells for 24 h (non‐shaded columns), and with 4T1 cells that were pre‐treated with the products of HAase and HA encapsulated PBS, Gal, NEU, SLO, SLO‐Gal, SLO‐NEU, or SLO‐Gal&SLO‐NEU for 24 h (shaded columns). Statistical analysis was performed by unpaired two‐tailed *t*‐tests (^**^ *p* < 0.01; ^***^ *p* < 0.001), n = 3.

The apoptosis or necrosis of tumor cells resulted from immune‐killing was further investigated with flow cytometric analysis by bicolor staining with Annexin V‐FITC and propidine iodide.^[^ ^23^ ^]^ In the absence of NK cells, all of the PBS, Gal, NEU, SLO, SLO‐Gal, SLO‐NEU, or SLO‐Gal&SLO‐NEU treated 4T1 cells maintained >90% in the viable region (Figure S9, Supporting Information), indicating that these treatments could not bring obvious apoptosis of tumor cells. However, after these treated 4T1 cells were further incubated with NK cells at the optimal ratio of 1:10 for 24 h, the cells in the viable region obviously decreased, and the lowest maintaining occurred in SLO‐Gal&SLO‐NEU treatment (**Figure** **3**a), indicating the maximum apoptosis and necrosis of tumor cells due to the immune‐killing, which was consistent to the CCK8 assay.

In NK cell‐based immunotherapy, the immune‐killing could be attributed to the secretion of cytokines from NK cells.^[^ ^10^ ^]^ The cytokines, including IFN‐γ, TNF‐α, IL‐2, perforin, and GrB, secreted from NK cells during the triply enhanced immune‐killing (Figure 3b) were analyzed by ELISA. All these cytokines exhibited an obvious in‐turn increase after NK cells were incubated with Gal, NEU, SLO, SLO‐Gal, SLO‐NEU, or SLO‐Gal&SLO‐NEU treated 4T1 cells (Figure 3c–g, non‐shaded columns), which indicated that the integration of Gal‐introduction, SA‐cleavage, and perforation exhibited the maximum enhancement in the in vitro immune‐killing.

### 2.4 Performance of TEID

**Figure 4:**
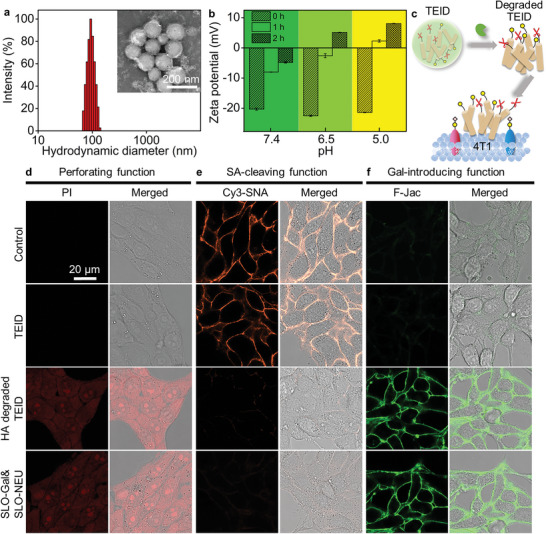
Characterization, degradation and triple functions of TEID. a) Dynamic light scattering (DLS) measurement of hydrodynamic size of TEID. Inset: TEM image of TEID. b) Zeta potentials of TEID after incubated with HAase at pH 5.0, 6.5, and 7.4 for different times. c) Schematic illustration of SLO‐Gal and SLO‐NEU release from TEID by HAase degradation to perform triple functions. d,e) CLSM images of 4T1 cells incubated with PBS (Control), TEID, HA degraded TEID, and SLO‐Gal&SLO‐NEU, and then stained with PI to verify perforating function (d) and Cy3‐SNA to verify SA‐cleaving function (e). f) CLSM images of GD pre‐treated 4T1 cells incubated with PBS (Control), TEID, HA degraded TEID, and SLO‐Gal&SLO‐NEU, and then stained with F‐Jac to verify Gal‐introducing function.

The encapsulation of SLO‐Gal&SLO‐NEU in an HA cross‐linked shell could avoid the denaturation of SLO‐Gal and SLO‐NEU in plasma and achieve tumor targeting through the specific recognition of HA to CD44 receptor on the tumor cell surface.^[^ ^18^, ^24^ ^]^ The synthesized TEID displayed a uniform spheroid structure with a diameter of ≈105 nm and a hydrodynamic size distribution narrower than those of previously reported HA structures^[^ ^18^, ^24^ ^]^(**Figure** **4**a). To verify the degradation of HA shell by HAase in the tumor microenvironment,^[^ ^18^ ^]^ the TEID was incubated with HAase at different pHs for different times and subjected to Zeta potential analysis. The mixtures incubated at pH 5.0 and 6.5 showed the change of Zeta potential from negative to positive value after incubation for 1 and 2 h (Figure 4b), respectively, while the Zeta potentials of TEID at these pHs did not change (Figure S10, Supporting Information), indicating the degradation of TEID to release positively charged SLO‐Gal and SLO‐NEU at low pHs. As the in vivo tumor microenvironment was weakly acidic (≈pH 6.5),^[^ ^24a^ ^]^ the HA shell of TEID could be quickly degraded by HAase to release SLO‐Gal and SLO‐NEU.

The function maintenance of the SLO‐Gal and SLO‐NEU released from TEID was investigated by treating the tumor cells with TEID, HA degraded TEID (Figure 4c), or the mixture of SLO‐Gal and SLO‐NEU, and then staining them with PI, Cy3‐SNA, or F‐Jac. In the absence of HA degradation, the TEID treated cells showed the same images as control cells (Figure 4d–f; Figure S11, Supporting Information), indicating the efficient isolation of SLO‐Gal and SLO‐NEU by HA shell. By contrary, the degradation of HA led to obvious fluorescence of PI, fluorescein, and Cy3, as those treated directly with the mixture of SLO‐Gal and SLO‐NEU, demonstrating the efficient performance of perforating, SA‐cleaving, and Gal‐introducing functions of the released SLO‐Gal and SLO‐NEU from TEID. The function maintenance was also demonstrated by the changes of PI, Cy3‐SNA, and F‐Jac fluorescence signals in flow cytometric analysis (Figure S12, Supporting Information).

The secretion of cytokines from NK cells was further examined with ELISA analysis after incubating 4T1 cells with PBS, TEID, or HAase degraded TEID, and then NK cells. Both PBS and TEID and then NK cells treated 4T1 cells showed the same low levels of cytokines (Figure S13, Supporting Information), indicating the excellent protective capacity of the HA shell to TEID, which limited the functions of encapsulated SLO‐Gal and SLO‐NEU. After the TEID was degraded by HAase, the released SLO‐Gal and SLO‐NEU could be anchored on 4T1 cells to perform the three functions, which led to the secretion of cytokines from NK cells, and thus significantly increased the levels of the cytokines. Moreover, the increases were significantly greater than those treated with the degraded products of HAase and HA encapsulated PBS, Gal, NEU, SLO, SLO‐Gal, or SLO‐NEU (Figure 3c–g, shaded columns), demonstrating the triply enhanced secretion of cytokines, demonstrating the triply enhanced secretion of cytokines, which promised the application of TEID in the in vivo immunotherapy.

### 2.5 In Vivo Immunotherapy by TEID

**Figure 5:**
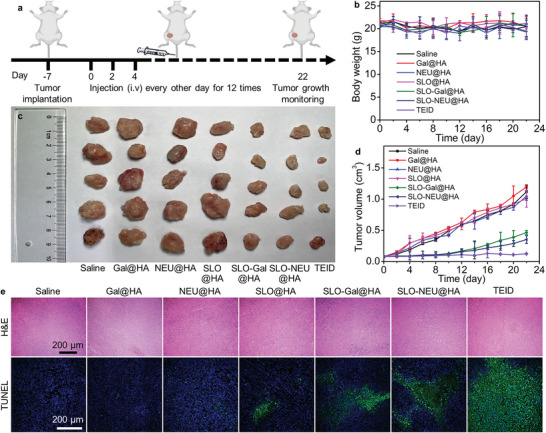
Triply enhanced immunotherapy with TEID on tumor‐bearing mice. a) Schematic illustration for implantation and treatment of 4T1 tumor‐bearing mice. b) Photo of tumor tissues dissected from 4T1 tumor‐bearing mice after injecting saline, Gal@HA, NEU@HA, SLO@HA, SLO‐Gal@HA, SLO‐NEU@HA, and TEID every other day for 12 times. c,d) Variation of body weight (c) and tumor volume (d) of 4T1 tumor‐bearing mice during injection. e) Histology and CLSM images of sectioned tumor tissues after H&E and TUNEL staining.

To demonstrate the triply enhanced in vivo immunotherapy by TEID, seven groups of the 4T1 tumor xenograft mice with tumor volume of ≈80 mm^3^ received intravenous injections of saline, HA encapsulated Gal (Gal@HA), NEU (NEU@HA), SLO (SLO@HA), SLO‐Gal (SLO‐Gal@HA), SLO‐NEU (SLO‐NEU@HA), and TEID every other day for 22 days, respectively (**Figure** **5**a). The body weight of all mice did not exhibit discernible difference during the treatment (Figure 5b), and their heart, liver, spleen, lung, and kidney did not also show abnormalities in the pathological observation (Figure S14, Supporting Information), indicating the negligible side effects under these treatments. The tumor volumes of the mice treated with Gal@HA, NEU@HA, and SLO@HA exhibited tiny variations comparing to these treated with saline (Figure 5c,d). In contrast, the mice treated with SLO‐Gal@HA or SLO‐NEU@HA exhibited a certain degree of inhibition to tumor volume, which could be attributed to the anchoring of SLO‐Gal or SLO‐NEU on tumor cells in tumor microenvironment to improve the NK cell‐based immune‐killing through the perforating and Gal‐introducing or SA‐cleaving functions, respectively. The mice treated with TEID exhibited the minimum volume, indicating the enhanced in vivo immunotherapy due to the integration of in situ dual glycan reforming with perforation. The pathological states of tumor tissues dissected from each treated group were assessed by hematoxylin and eosin (H&E) and terminal deoxynucleotidyl transferase dUTP nick end labeling (TUNEL) assay. The sectioned tissue from TEID treated mouse exhibited the largest necrotic area compared to those from SLO‐Gal@HA or SLO‐NEU@HA treated mouse (Figure 5e), which was consistent to the changes of tumor volumes. Thus, the TEID exactly exhibited the maximum enhancement in in vivo immunotherapy.

**Figure 6:**
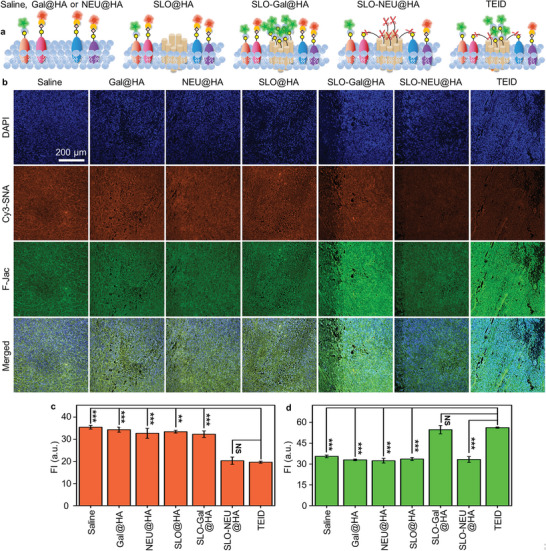
Verification of triple functions on tumor tissue for immunotherapy with TEID. a) Schematic illustration of sectioned tumor tissues after treated with saline, Gal@HA, NEU@HA, SLO@HA, SLO‐Gal@HA, SLO‐NEU@HA, or TEID. b) CLSM images of DAPI, Cy3‐SNA, and F‐Jac stained tumor tissue slices from tumor‐bearing mice after therapy with saline, Gal@HA, NEU@HA, SLO@HA, SLO‐Gal@HA, SLO‐NEU@HA, or TEID. c,d) Fluorescence intensities (FI) of Cy3‐SNA (c) and F‐Jac (d) from (b). Statistical analysis was performed by unpaired two‐tailed *t*‐tests (^**^ *p* <0.01; ^***^ *p* <0.001; NS, not significant), n = 3.

To further validate the enhancing mechanisms of TEID in in vivo immunotherapy, the tumor tissues sectioned from seven treated mouse groups were respectively stained with Cy3‐SNA and F‐Jac (**Figure** **6**a). The tissue slices from the mice treated with Gal@HA, NEU@HA, and SLO@HA exhibited similar fluorescence of Cy3‐SNA and F‐Jac compared to those treated with saline (Figure 6b–d). In contrast, the tissue slices from SLO‐Gal@HA or SLO‐NEU@HA treated mice exhibited a significant increase of F‐Jac fluorescence or a decrease of Cy3‐SNA fluorescence (Figure 6b–d). Besides, the tissue slice from the TEID treated mouse exhibited a simultaneous decrease of Cy3‐SNA and an increase of F‐Jac fluorescence (Figure 6b–d). These results indicated that the perforation of SLO and the introduction of Gal and NEU to tumor tissue for performing dual glycan reforming played critical roles in in vivo immunotherapy.

## 3 Conclusion

In summary, a triply enhanced immunotherapy strategy is proposed with a designed TEID to integrate in situ dual glycan reforming with perforation of exogenous perforating molecule on cell surface. The TEID can be conveniently prepared by encapsulating SLO‐Gal&SLO‐NEU in HA shell to achieve targeted delivery, HAase‐induced degradation in tumor microenvironment, and easily anchoring of SLO‐Gal and SLO‐NEU on tumor cells to perform the Gal‐introduction, SA‐cleavage and perforation in vivo, which exhibits the significant enhancement of immunotherapeutic efficacy of the tumors through simultaneously boosting NK cell activation, weakening immune cell inhibition and promoting the delivery of NK cell‐secreted cytokines. The proposed strategy provides a significant and promising route for clinical immunotherapy of tumors.

## Conflict of Interest

The authors declare no conflict of interest.

## Supporting information

Supporting Information

## Data Availability

The data that support the findings of this study are available from the corresponding author upon reasonable request.
