# Correction to “Triply Enhanced Immunotherapy via Dual Glycan Reforming Integrated with Perforation”

**DOI:** 10.1002/advs.202309028

**Published:** 2024-02-11

**Authors:** Yuanjiao Yang, Yuru Wang, Zhicong Chao, Yuhui Yang, Yanyun Fang, Ying Liu, Lin Ding, Yunlong Chen, Huangxian Ju

*Adv. Sci*. **2023**, 2304971

https://doi.org/10.1002/advs.202304971

Pages 3, 6 and 7. We noticed misuses of the CLSM images in Figure 2b line 3 for SLO‐Gal and Figure 4f lines 1 and 4 for Control and SLO‐Gal&SLO‐NEU, respectively, and the H&E staining image in Figure 5e line 1 column 4 for SLO@HA.

**Figure 2:**
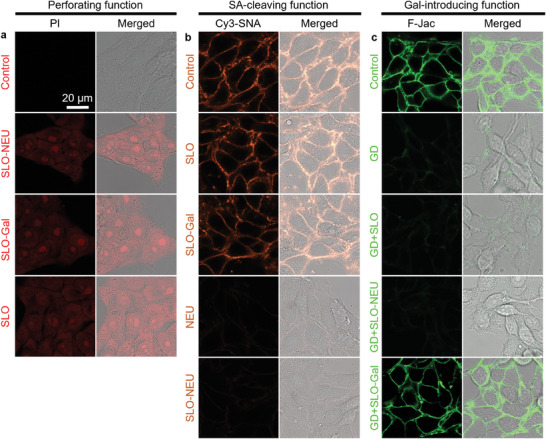
Verification of triple functions of SLO‐Gal and SLO‐NEU on 4T1 cells. a,b) CLSM images of 4T1 cells after incubated with PBS (Control), SLO, SLO‐Gal, or SLO‐NEU and then stained with PI to verify perforating function (a), and with PBS (Control), SLO, SLO‐Gal, NEU or SLO‐NEU and then stained with Cy3‐SNA to verify the SA‐cleaving function (b). c) CLSM images of 4T1 cells and GD per‐treated 4T1 cells after incubated with PBS (Control and GD), SLO, SLO‐Gal, or SLO‐NEU and then stained with F‐Jac to verify Gal‐introducing function.

**Figure 4:**
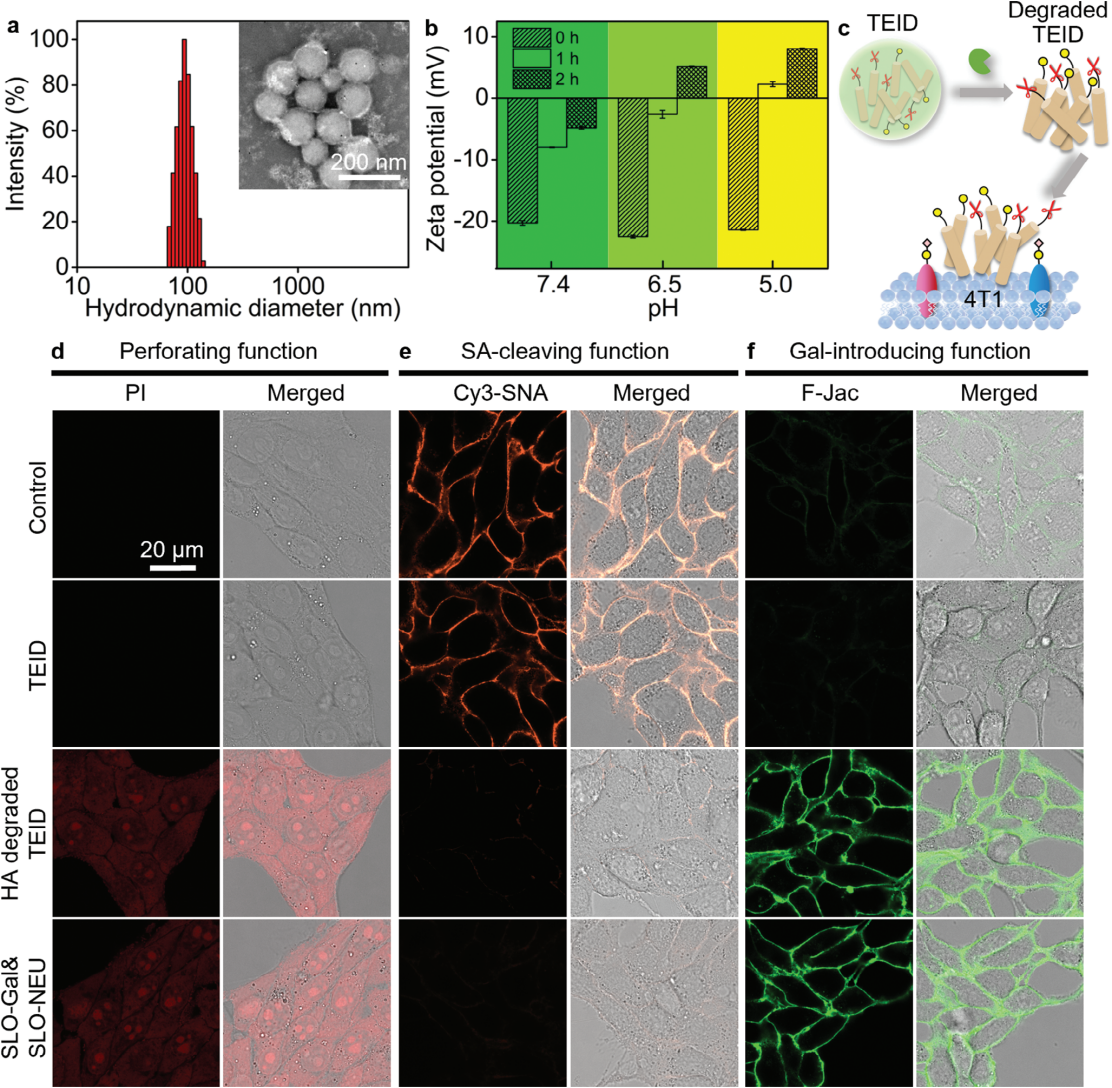
Characterization, degradation and triple functions of TEID. a) Dynamic light scattering (DLS) measurement of hydrodynamic size of TEID. Inset: TEM image of TEID. b) Zeta potentials of TEID after incubated with HAase at pH 5.0, 6.5, and 7.4 for different times. c) Schematic illustration of SLO‐Gal and SLO‐NEU release from TEID byHAase degradation to perform triple functions. d,e) CLSM images of 4T1 cells incubated with PBS (Control), TEID, HA degraded TEID, and SLO‐Gal&SLO‐NEU, and then stained with PI to verify perforating function (d) and Cy3‐SNA to verify SA‐cleaving function (e). f) CLSM images of GD pre‐treated 4T1 cells incubated with PBS (Control), TEID, HA degraded TEID, and SLO‐Gal&SLO‐NEU, and then stained with F‐Jac to verify Gal‐introducing function.

**Figure 5:**
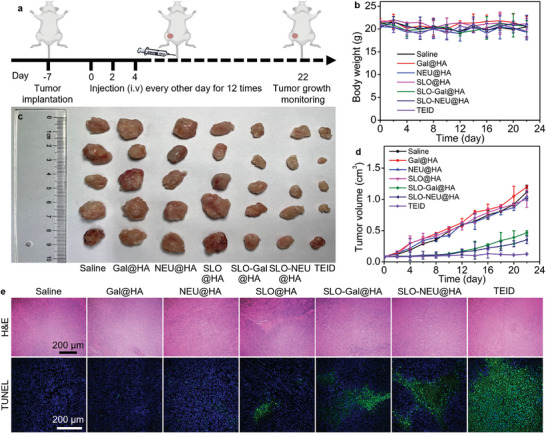
Triply enhanced immunotherapy with TEID on tumor‐bearing mice. a) Schematic illustration for implantation and treatment of 4T1 tumor‐bearing mice. b) Photo of tumor tissues dissected from 4T1 tumor‐bearing mice after injecting saline, Gal@HA, NEU@HA, SLO@HA, SLO‐Gal@HA, SLO‐NEU@HA, and TEID every other day for 12 times. c,d) Variation of body weight (c) and tumor volume (d) of 4T1 tumor‐bearing mice during injection. e) Histology and CLSM images of sectioned tumor tissues after H&E and TUNEL staining.

Pages 7 and 12 in Supporting Information. There are misuses of the CLSM images in Figure S3c lines 2 and 3 for GD and GD+SLO, respectively, and the H&E staining images in Figure S14 line 1 columns 4, 6 and 7 for SLO@HA, SLO‐NEU@HA and TEID respectively, line 2 column 7 for TEID, line 3 column 7 for TEID, line 4 columns 6 and 7 for SLO‐NEU@HA and TEID respectively, and line 5 column 4 for SLO@HA.

These images need to be corrected, but the results and conclusions in the published Article are not changed. The corrected Figures 2, 4 and 5 are presented below. Figures S3 and S14 have been corrected in the updated Supporting Information.

## Supporting information

Supporting Information

