# Supplementary material for: Triply Enhanced Immunotherapy via Dual Glycan Reforming Integrated with Perforation
Source: Adv Sci (Weinh). 2023 Oct 23;11(2):2304971. doi: 10.1002/advs.202304971 (PMC10787084; doi:10.1002/advs.202304971)
Supplement: Supplementary file 1 — Supporting Information [file ADVS-11-2304971-s001.pdf]

ADVANCED SCIENCE
Open Access

Supporting Information

for *Adv. Sci.*, DOI 10.1002/advs.202304971

Triply Enhanced Immunotherapy via Dual Glycan Reforming Integrated with Perforation

Yuanjiao Yang, Yuru Wang, Zhicong Chao, Yuhui Yang, Yanyun Fang, Ying Liu, Lin Ding, Yunlong Chen and Huangxian Ju**

Triply Enhanced Immunotherapy via Dual Glycan Reforming Integrated with Perforation

Yuanjiao Yang, Yuru Wang, Zhicong Chao, Yuhui Yang, Yanyun Fang, Ying Liu, Lin Ding, Yunlong Chen,* and Huangxian Ju*

Abstract: The enhancement of immunotherapy is an emerging direction to develop highly effective and practical cancer therapeutic methods. Here a triply enhanced immunotherapy drug (TEID) is designed for ingeniously integrating in situ dual glycan reforming with perforation on cell membrane. The TEID is composed of galactose and neuraminidase conjugated streptolysin O (SLO-Gal and SLO-NEU), which are encapsulated in a hyaluronic acid (HA) shell for targeted recognition to tumor tissue via cell surface CD44. After targeted delivery and HAase mediated degradation in tumor region, the TEID releases SLO-Gal and SLO-NEU, which can easily anchor Gal and NEU on tumor cell membrane via the perforation of SLO to perform dual glycan reforming for the introduction of Gal and the cleavage of sialic acid. The former can activate immune cells to secret cytokines for immune-killing, and the latter can weaken the immune inhibition to improve the immunotherapeutic efficacy. Meanwhile, the perforation of SLO can promote the delivery of cytokines into the tumor cells to further enhance the efficacy. The designed triply enhanced immunotherapy strategy opens a significant and promising route to promote clinical immunotherapy of cancer.

Table of Contents

Experimental procedures 3-5

Materials and Reagents 3

Apparatus 3

Synthesis of SLO-NEU, SLO-Gal and TEID 3

CLSM cell imaging and flow cytometric analysis of perforating, SA-cleaving and Gal-introducing functions 4

CLSM imaging of perforating function on NK cells 4

CCK-8 assay and flow cytometric analysis of the cytotoxicity of SLO-Gal and SLO-NEU 4

CCK-8 assay of cytotoxicity 4

Quantification of cytokines secreted from NK cells 4

Animals and tumor implantation models 5

In vivo enhanced cancer immunotherapy 5

Triple functions on tissue slices 5

Supporting Figures 6-12

Figure S1. Mass spectra of 4AC-Gal-N_3, Gal-N_3, SLO, SLO-DBCO, SLO-Gal, NEU, and NEU-N_3 6

Figure S2. The SA cleaving performance of NEU and SLO-NEU 6

Figure S3. Verification of the triple functions of SLO-Gal and SLO-NEU on MCF-7 cells 7

Figure S4. CLSM images of PI stained NK cells after incubation with SLO, SLO-NEU or SLO-Gal 7

Figure S5. The flow cytometric analysis of the triple functions of SLO-Gal and SLO-NEU 8

Figure S6. Cell viability of tumor cells after enhanced immune-killing with NK cells 8

Figure S7. Cell viability of tumor cells after killing with PBS (control) or different effectors (T cell, PBMC and NK cell) 9

Figure S8. Cell viability of tumor cells after immune-killing with NK cells at different ratios of NK to tumor cells 9

Figure S9. Flow cytometric scatter plots of the 4T1 cells treated with different components in the absence of NK cells 9

Figure S10. Zeta potentials of TEID at different pHs for different times 10

Figure S11. CLSM verification of the triple functions of TEID and the degraded TEID on MCF-7 cells 10

Figure S12. Flow cytometric analysis of the triple functions of TEID and the degraded TEID 11

Figure S13. ELISA analysis of the secretion of cytokines from NK cells after incubation with TEID and the degraded TEID 11

Figure S14. The side effects of TEID in the immunotherapy of tumor-bearing mice 12

References 12

Experimental Procedures

Materials and Reagents. Streptolysin O (SLO), propidium iodide (PI), dibenzocyclooctyne-sulfo-N-hydroxysuccinimidyl ester (DBCO-sulfo-NHS ester), azido-dPEG8-NHS ester (N_3-PEG8-NHS), bovine serum albumin (BSA), β-galactosidase (GD), poly-L-lysine, 2'-(4-Methylumbelliferyl)-α-D-N-acetylneuraminic acid (MuNeuNAc) and 4-methylumbelliferone were purchased from Sigma-Aldrich Inc. (USA). MCF-7, 4T1 and T cells (human T lymphocytes), trypsin, phosphate buffered saline (PBS), RPMI-1640 cell culture medium and Hank's balanced salt solution (HBSS) were supplied by KeyGen Biotech. Co. Ltd. (China). Cy3 labelled Sambucus Nigra Lectin (Cy3-SNA), and fluorescein labelled Jacalin (F-Jac) were from Vector Laboratories (USA). Human NK-92 cells and IL-2 protein were from Procell Life Science & Technology Co., Ltd. (China). Hyaluronic acid (HA), methacrylic anhydride (MA), NaOH, hyaluronidase (HAase), ethanol, acetone, xylene, 2-hydroxy-4'-(2-hydroxyethoxy)-2-methylpropiophenone (Irgacure 2959), sodium methoxide, methanol, D-galactose (Gal) and 2-[2-(2-azidoethoxy)ethoxy]ethyl 2,3,4,6-tetra-O-acetyl-D-galactopyranoside (4AC-Gal-N_3) were from Shanghai Macklin Biochemical Co., Ltd. (China). DAPI staining solution and neutral resin were from Beyotime Biotechnology (China). DOWEX(R) 50 WX8-200 ion-exchange resin was obtained from Alfa Aesar Inc. (USA). Glycerol dimethacrylate, phosphotungstic acid and α2-3,6,8 neuraminidase A (NEU) from *Clostridium perfringens* were obtained from Shanghai Aladdin Ltd. (China). Human peripheral blood lymphocyte isolate fluid (endotoxin < 0.25 EU) was purchased from Tian Jin Hao Yang Biological Manufacture Co., Ltd. (China). Cell Counting Kit (CCK-8) was obtained from Shanghai Dojindo China Co., Ltd. (China). Enzyme-linked immunosorbent assay (ELISA) kits for IFN-γ, TNF-α, IL-2, perforin and Granular enzyme B (GrB) were from Shanghai MEIMIAN Ltd. (China). Annexin V-FITC apoptosis detection kit was from Yeasen Co., Ltd. (China). All solutions were prepared using ultrapure water (≥18 MΩ, Milli-Q, Millipore).

Apparatus. Transmission electron microscopic (TEM) images were obtained on a JEM-2100 transmission electron microscope (JEOL Ltd., Japan). Confocal fluorescence micrographs were acquired on a TCS SP8 confocal laser scanning microscope (CLSM) (Leica, Germany). The UV-vis absorption spectra were recorded using a UV-vis spectrophotometer (Nanodrop-2000C, Nanodrop, USA). Flow cytometric analysis was gained on a CytoFLEX flow cytometer (Beckman-Coulter, USA). Zeta potential analysis was performed on a Zetasizer (Nano-Z, Malvern, UK). Dynamic light scattering (DLS) measurements were performed on a 90 Plus/BI-MAS equipment (Brook haven, USA). CCK8 assays were performed on a Varioskan Flash spectral scanning multimode reader (Thermo Fisher Scientific, USA).

Synthesis of SLO-NEU, SLO-Gal and TEID. SLO-DBCO was firstly prepared by mixing SLO (0.55 mM = 3.0×10^7 U mL^{-1}, one unit will cause 50% lysis of 50 μL of a 2% human red blood cell suspension in phosphate buffered saline, pH 7.4, at 37 °C for 30 min) and DBCO-sulfo-NHS ester (47 mM) to react at room temperature for 2 h, and then filtrating the mixture with 30 kDa MWCO membrane. Similarly, NEU-N_3 was prepared by mixing NEU (0.02 mM) and N_3-PEG8-NHS (0.1 mM) to react at room temperature for 2 h. Meanwhile, azide labelled galactose (Gal-N_3) was prepared by stirring the mixture of 4AC-Gal-N_3 (10 mM) and sodium methoxide (2.5 mM) in dry methanol at room temperature overnight, and then filtering with DOWEX(R) 50 WX8-200 ion-exchange resin. MS (ESI, methanol): m/z = 360 $[M + Na]^+$.

The SLO-NEU or SLO-Gal conjugates were obtained by mixing SLO-DBCO (1 mM) with NEU-N_3 (0.01 mM) or Gal-N_3 (10 mM) at room temperature for 2 h, followed by ultrafiltration with 100 kDa or 30 kDa MWCO membrane to remove the excess SLO-DBCO or Gal-N_3, respectively. The NEU activity of SLO-NEU was evaluated by incubating 1 mM MuNeuNAc with NEU (1 nM) or SLO-NEU (1 nM) at 37 °C for various times to determine the fluorescence of released methylumbelliferone at 445 nm with 365 nm excitation.

The TEID was prepared by encapsulating SLO-Gal and SLO-NEU in HA shell following a previous method.[S1] Briefly, 0.16 mL MA was dropwise added in 10 mL 0.02 g mL^{-1} HA at 4 °C, and the solution was adjusted to pH 8-9 with 5.0 M NaOH. After continuously stirring for 24 h, the resulting polymer was precipitated with acetone, washed with ethanol, re-dissolved in water and lyophilized to obtain methacrylated HA (m-HA). Afterward, 100 U mL^{-1} SLO-NEU, 100 U mL^{-1} SLO-Gal (U mL^{-1} is the concentration of SLO), 0.07 mg glycerol dimethacrylate and Irgacure

2959 (0.1%, w:v) were sequentially added into 200 μL stirring m-HA solution (0.35 mg mL^{-1}) to perform radical polymerization by exposing the solution to ultraviolet radiation (20 mV cm^{-2}) for 60 s. The resulting TEID were obtained by centrifuging with 3 kDa MWCO membrane to remove the excessive crosslinker and initiator. The Gal@HA, NEU@HA, SLO@HA, SLO-Gal@HA and SLO-NEU@HA were prepared with the same procedure.

CLSM cell imaging and flow cytometric analysis of perforating, SA-cleaving and Gal-introducing functions. After MCF-7 or 4T1 cells were seeded on confocal dishes overnight and washed three times with HBSS, the cells were incubated with PBS, 200 U mL^{-1} SLO, SLO-NEU, SLO-Gal or 0.1 μM NEU at 37 ºC for 30 min, and stained with PI or Cy3-SNA (10 μg mL^{-1}) at 4 ºC for 60 min to verify the perforating function of SLO or SA-cleaving function of SLO-NEU with CLSM imaging under 535 nm for PI channel or 543 nm for Cy3 channel, respectively. The Gal-introducing function of SLO-Gal was verified at 495 nm by incubating seeded MCF-7 or 4T1 cells with 20 U mL^{-1} GD at 37 ºC for 60 min to obtain GD per-treated cells, and then incubating them with PBS, 200 U mL^{-1} SLO, SLO-Gal or SLO-NEU at 37 ºC for 30 min to stain with F-Jac (10 μg mL^{-1}) at 4 ºC for 60 min.

The function verification of HA degraded TEID was performed by incubating TEID with 0.4 mg mL^{-1} HAase at pH 6.5 at 37 ºC for 2 h, and then with the seeded MCF-7 or 4T1 cells at 37 ºC for 30 min to stain with PI or Cy3-SNA, or with β-galactosidase (GD) treated MCF-7 or 4T1 cells at 37 ºC for 30 min stain with F-Jac. The imaging signals were collected from 555 to 625 nm for PI channel, 555 to 640 nm for Cy3 channel and 510 to 600 nm for fluorescein channel. Flow cytometric analysis was performed by collecting these stained cells with centrifugation at 1,000 rpm for 3 min and resuspending them in 500 μL PBS.

CLSM imaging of perforating function on NK cells. After NK cells were incubated with PBS, 200 U mL^{-1} SLO, SLO-NEU or SLO-Gal at 37 ºC for 30 min, the cells were washed three times with HBSS and stained with PI (10 μg mL^{-1}) at 4 ºC for 60 min. The cells were then seeded on poly-L-lysine treated confocal dishes for 10 min and imaged by CLSM to collect the emission signals from 555 nm to 625 nm under 535 nm excitation.

CCK-8 assay and flow cytometric analysis of the cytotoxicity of SLO-Gal and SLO-NEU. After MCF-7 cells or 4T1 cells seeded on dishes were incubated with PBS, 0.1 μM Gal, 0.1 μM NEU, 200 U mL^{-1} SLO, SLO-Gal, SLO-NEU, or the mixture of 100 U mL^{-1} SLO-Gal and SLO-NEU at 37 ºC for 30 min, they were incubated with NK cells with a ratio of 1:1 at 37 °C for 8 h, 12 h, 24 h and 48 h, to analyze the cell viability through CCK8 assay, respectively. The optimal ratio of NK to cancer cells was obtained by incubating 10000 seeded MCF-7 or 4T1 cells with the mixture of 100 U mL^{-1} SLO-Gal and SLO-NEU at 37 ºC for 30 min, and then incubating them with different amounts of NK cells at 37 °C for 24 h to detect the cell viability.

The flow cytometric analysis of the cytotoxicity was performed by incubating the seeded 4T1 cells with PBS, 0.1 μM Gal, 0.1 μM NEU, 200 U mL^{-1} SLO, SLO-Gal, SLO-NEU, or the mixture of 100 U mL^{-1} SLO-Gal and SLO-NEU at 37 ºC for 30 min, and then with NK cells at 37 °C for 24 h to stain with AnnexinV-FITC/PI.

CCK-8 assay of cytotoxicity. Peripheral blood mononuclear cells (PBMCs) were isolated from human peripheral blood obtained from Nanjing Integrated Traditional Chinese and Western Medicine Hospital by density gradient separation with human peripheral blood lymphocyte isolate fluid. After MCF-7 cells or 4T1 cells seeded on dishes and incubated with the mixture of 100 U mL^{-1} SLO-Gal and SLO-NEU at 37 ºC for 30 min, the cells were respectively incubated with PBS or different effectors (T cells, PBMCs and NK cells) with the ratio of 1:1 at 37 °C for 24 h to detect the cell viability.

Quantification of cytokines secreted from NK cells. After the seeded 4T1 cells were incubated with PBS, 0.1 μM Gal and NEU, 200 U mL^{-1} SLO, SLO-Gal and SLO-NEU, the mixture of 100 U mL^{-1} SLO-Gal and SLO-NEU (U mL^{-1} is the concentration of SLO), HAase treated HA encapsulated PBS, Gal, NEU, SLO, SLO-Gal or SLO-NEU, or HAase treated TEID at 37 ºC for 30 min, and then with NK cells at 37 °C for 24 h,

the supernatant containing IFN-γ, TNF-α, IL-2, perforin and Granular enzyme B was collected by centrifugation at 1,000 rpm for 5 min to perform the ELISA quantification.

Animals and tumor implantation models. BALB/c was a laboratory-bred albino strain of the house mouse and one of the most widely used mouse strains across oncology and immunology. Six-week-old (18 g) pathogen-free female BALB/c mice were purchased from Keygen Biotech. Co. Ltd. (China), and treated according to the NIH guidelines for the care and use of laboratory animals (NIH Publication no. 85-23 Rev. 1985) and approved by the Jiangsu Administration of Experimental Animals with 230035 number of IACUC-2305004. To set up the 4T1 tumor-bearing mouse model, 2×10^6 4T1 cancer cells suspended in PBS were subcutaneously inoculated into the right flank of each mouse.

***In vivo* enhanced cancer immunotherapy.** After tumor volume reached about 80 mm^3, the 4T1 tumor-bearing BALB/c mice were randomly divided into 7 groups (n=5). The mice were intravenously injected in tail every other day for 22 days with 100 μL (1) saline, (2) 0.1 μM Gal@HA, (3) 0.1 μM NEU@HA, (4) 200 U mL^{-1} SLO@HA, (5) 200 U mL^{-1} SLO-Gal@HA, (6) 200 U mL^{-1} SLO-NEU@HA, and (7) TEID, respectively. Meanwhile, the tumor weights were recorded, the tumor sizes were measured, and the tumor volume (V) was calculated as $V = (L \times W^2)/2$, where L and W were the length and width of the tumor, respectively. At Day 22, the tumors, hearts, kidneys, spleens, lungs, and livers were dissected from the euthanized mice, fixed in the 4% paraformaldehyde solution, embedded in paraffin blocks, sliced into sections and stained with hematoxylin and eosin (H&E) to visualize with an optical microscope (Olympus BX51, Japan). The tumor slices were also performed TUNEL assay and CLSM imaging under 405 nm with signal collection from 430 to 500 nm for DAPI channel or 495 nm signal collection from 505 to 575 nm for FITC channel.

Triple functions on tissue slices. The verification of glycan reforming on tissue slices referenced the previous method.[S2] Paraffin slices of 7 groups of mice after immunotherapy were dewaxed in xylene for 2 h, and then hydrated in 100, 95, 90, 80, 75, 70 and 50% ethanol and H_2O for 3 min in turn. The hydrated tissue slices were incubated with 5% BSA at 37 °C for 1 h and washed with PBS three times (3 min each). Afterward, the tissue slices were stained with Cy3-SNA (10 μg mL^{-1}) and F-Jac (10 μg mL^{-1}) at 4 °C for 5 h, and then DAPI for 20 min. Finally, the tissue slices were sealed with neutral resin and imaged by CLSM under 405 nm for DAPI, 543 nm for Cy3 and 495 nm for fluorescein channels to collect the emission signals from 430 to 500 nm, 555 to 640 nm and 510 to 600 nm, respectively.

Supporting Figures

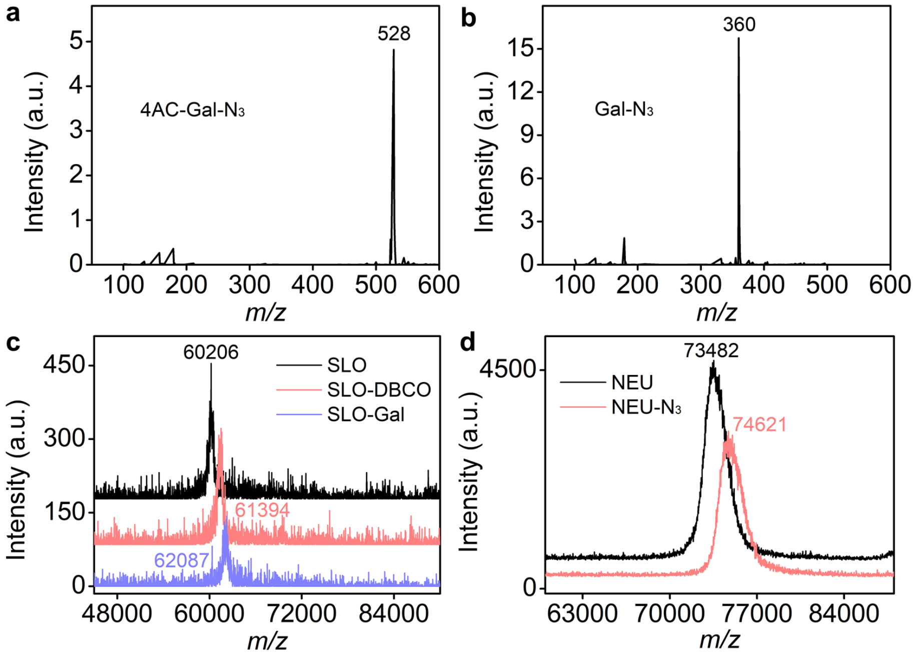

Figure S1. Mass spectra of 4AC-Gal-N_3, Gal-N_3, SLO, SLO-DBCO, SLO-Gal, NEU, and NEU-N_3. a, b) ESI-MS spectra of 4AC-Gal-N_3 (a) and Gal-N_3 (b). c, d) MALDI-TOF mass spectra of SLO, SLO-DBCO and SLO-Gal (c), and NEU and NEU-N_3 (d).

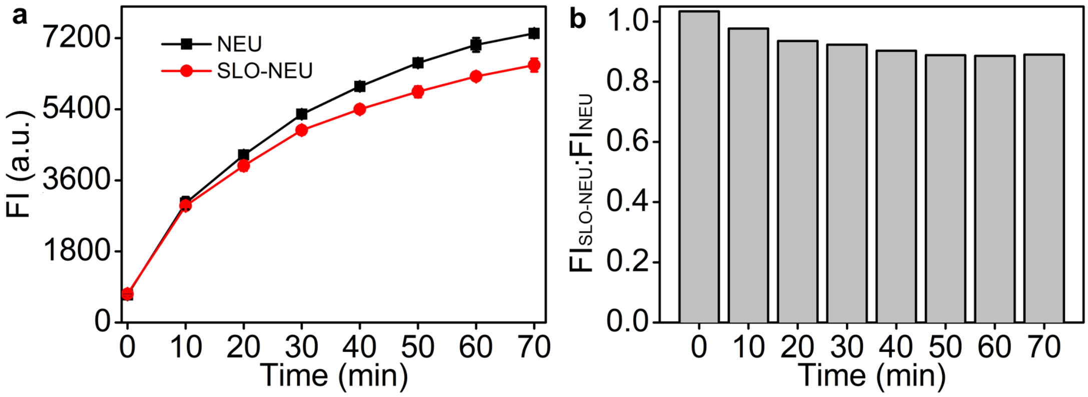

Figure S2. The SA cleaving performance of NEU and SLO-NEU. a) Fluorescence intensity (FI) of MuNeuNAc at emission wavelength of 445 nm after incubated with NEU or SLO-NEU at 37 ºC for different times. b) The FI ratio of MuNeuNAc after incubated with SLO-NEU to with NEU in (a). The excitation wavelength of released methylumbelliferone was 325 nm. The data error bars represent mean ± SD (n = 3).

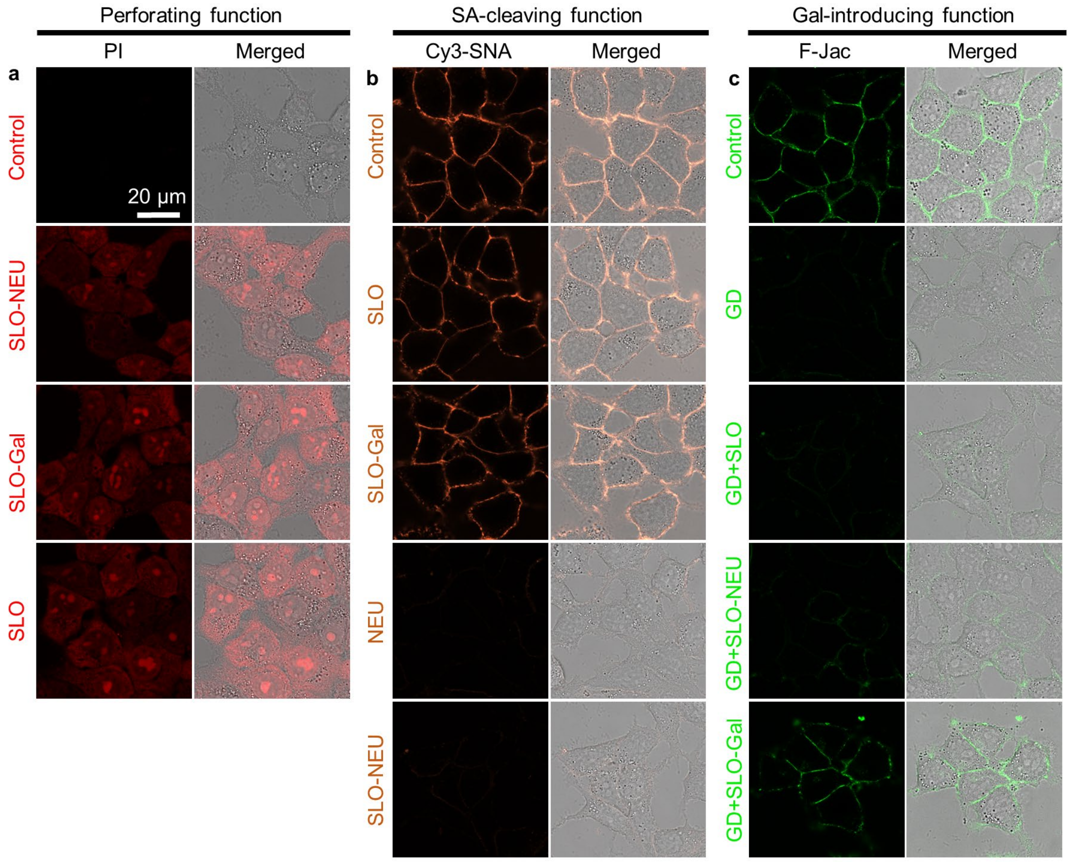

Figure S3. Verification of the triple functions of SLO-Gal and SLO-NEU on MCF-7 cells. a, b) CLSM images of MCF-7 cells after incubated with PBS (Control), SLO, SLO-Gal or SLO-NEU and then stained with PI to verify perforating function (a), and Cy3-SNA to verify SA-cleaving function (b). c) CLSM images of MCF-7 cells and GD per-treated MCF-7 cells after incubated with PBS (Control and GD), SLO, SLO-Gal or SLO-NEU and then stained with F-Jac to verify Gal-introducing function.

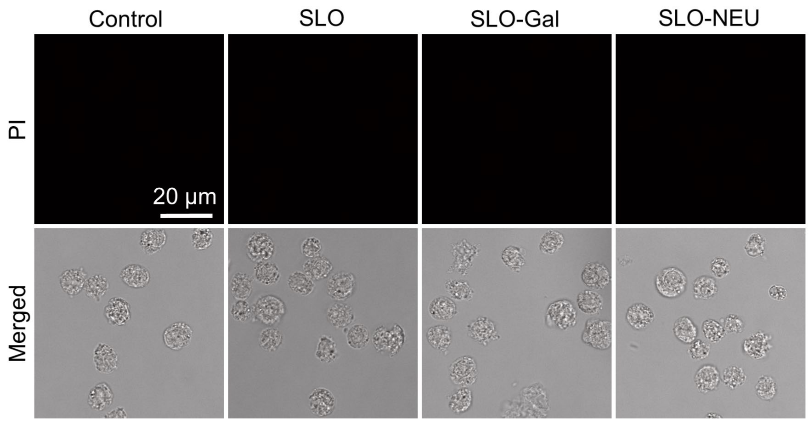

Figure S4. CLSM images of PI stained NK cells after incubation with SLO, SLO-NEU or SLO-Gal. CLSM images of PI stained NK cells after incubation with 200 U mL^{-1} SLO, SLO-NEU or SLO-Gal at 37 ℃ for 30 min.

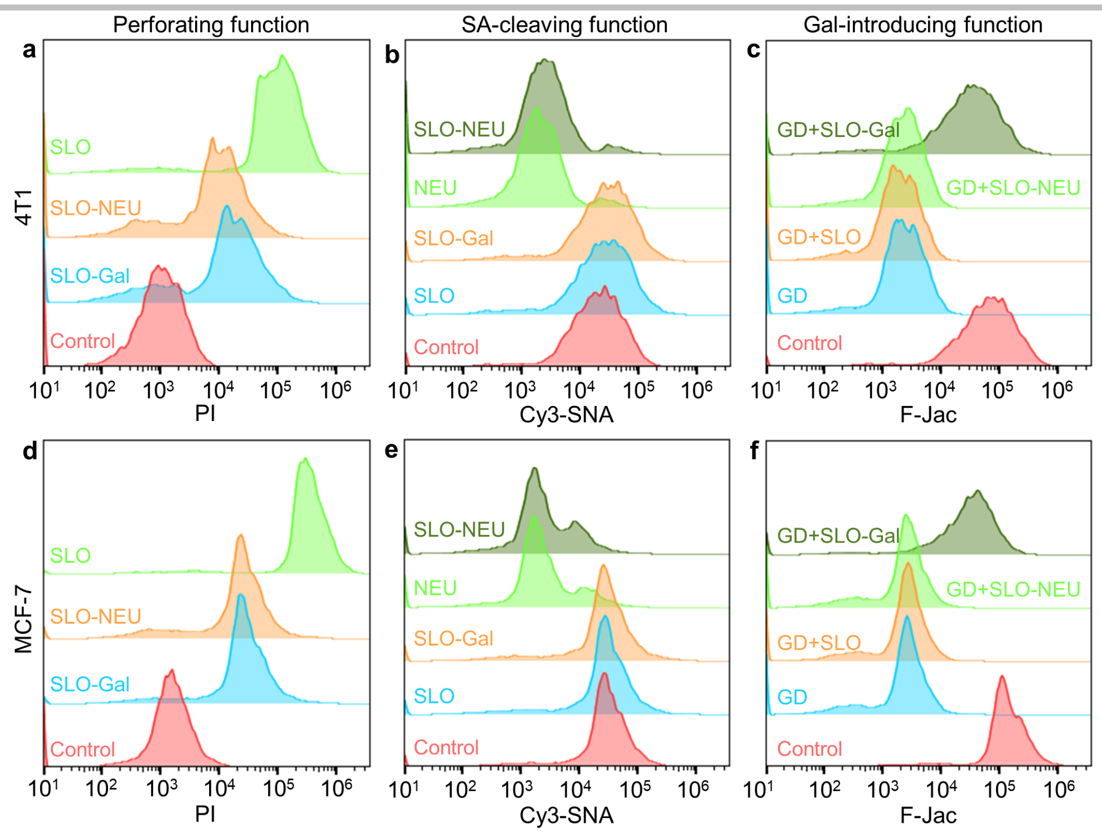

Figure S5. The flow cytometric analysis of the triple functions of SLO-Gal and SLO-NEU. a, b, d, e) Flow cytometric analysis of 4T1 or MCF-7 cells incubated with PBS (Control), SLO, SLO-NEU or SLO-Gal and then stained with PI to verify the perforating function (a, d), and with PBS (Control), SLO, SLO-Gal, NEU or SLO-NEU and then stained with Cy3-SNA to verify the SA-cleaving function (b, e). c, f) Flow cytometric analysis of 4T1 or MCF-7 cells and GD per-treated 4T1 or MCF-7 cells after incubated with PBS (Control and GD), SLO, SLO-Gal or SLO-NEU and then stained with F-Jac to verify Gal-introducing function.

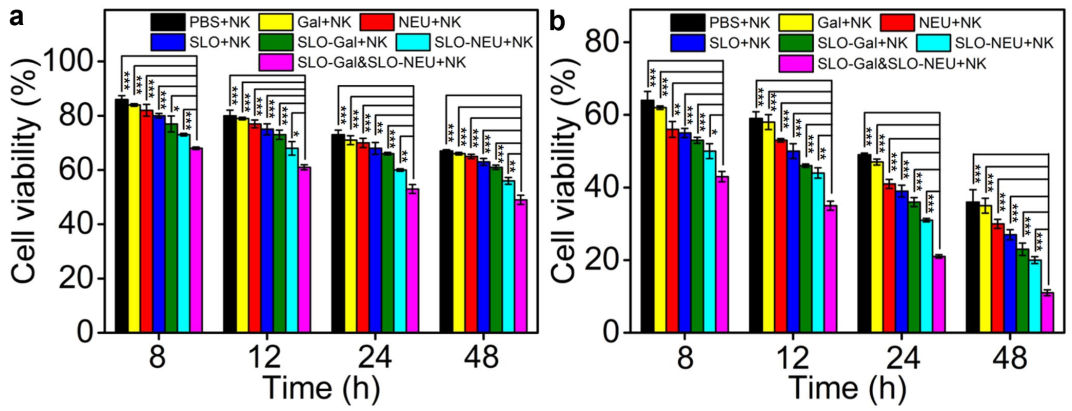

Figure S6. Cell viability of tumor cells after enhanced immune-killing with NK cells. Cell viability of 4T1 cells (a) and MCF-7 cells (b) after performing SLO-Gal and SLO-NEU enhanced immune-killing with NK cells for different times. Statistical analysis was performed by unpaired two-tailed t-tests (*p < 0.05; **p < 0.01; ***p < 0.001; NS, not significant), *n=3*.

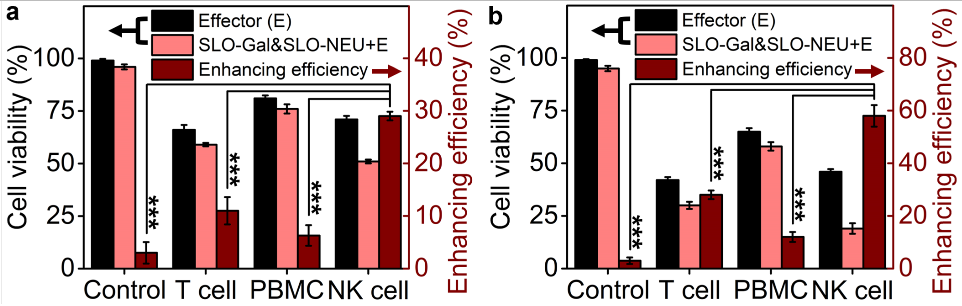

Figure S7. Cell viability of tumor cells after incubating with PBS (control) or different effectors (T cell, PBMC and NK cell). Cell viability of 4T1 cells (a) and MCF-7 cells (b) after performing common immune-killing (black columns), SLO-Gal and SLO-NEU enhanced immune-killing (pink columns) with different effectors. The enhancing efficiency (red columns) were calculated with ($Viability_E$ - $Viability_{SLO\text{-}Gal\ \&\ SLO\text{-}NEU\ +\ E}$) / $Viability_E$. Statistical analysis was performed by unpaired two-tailed t-tests (***p < 0.001), *n=3*.

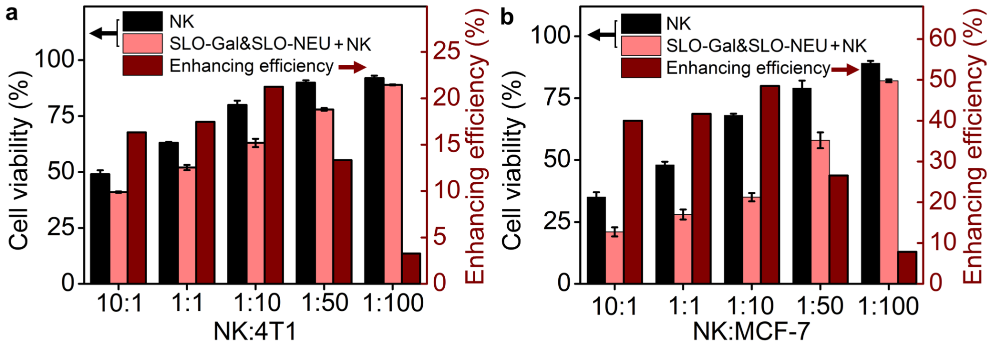

Figure S8. Cell viability of tumor cells after immune-killing with NK cells at different ratios of NK to tumor cells. Cell viability of 4T1 cells (a) and MCF-7 cells (b) after performing common immune-killing (black columns), SLO-Gal and SLO-NEU enhanced immune-killing (pink columns) with NK cells. The enhancing efficiency (red columns) at different ratios of NK to cancer cells was calculated with (ViabilityNK - ViabilitySLO-Gal & SLO-NEU + NK) / ViabilityNK. The error bars indicate mean ± SD (n = 3).

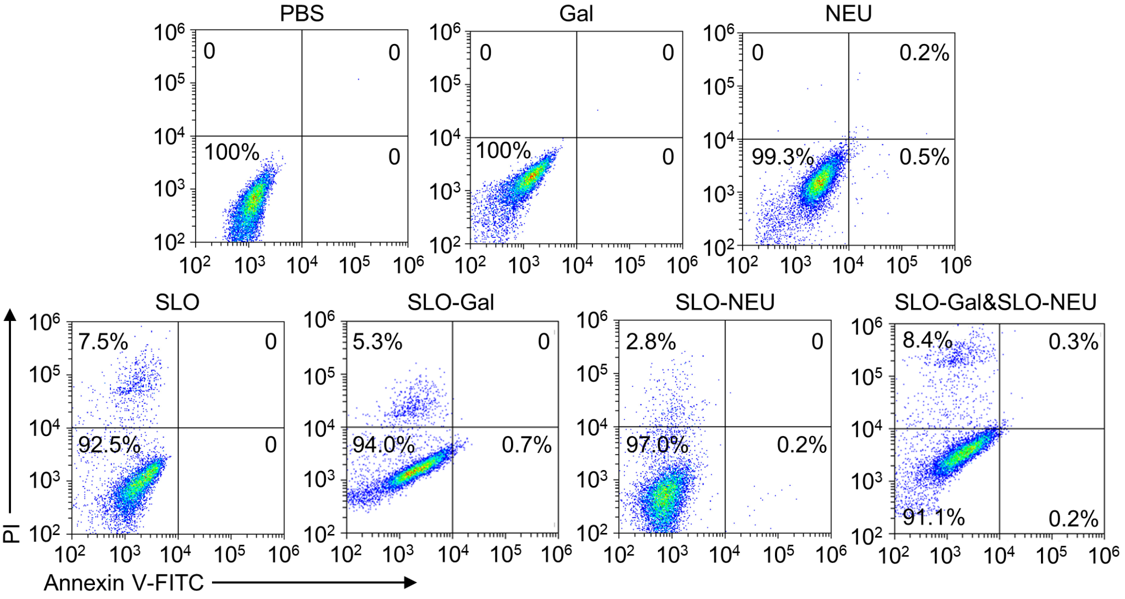

Figure S9. Flow cytometric scatter plots of the 4T1 cells treated with different components in the absence of NK cells. Flow cytometric scatter plots of 4T1 cells after incubation with Gal, NEU, SLO, SLO-Gal, SLO-NEU or the mixture of SLO-Gal and SLO-NEU and then staining with Annexin V-FITC and PI.

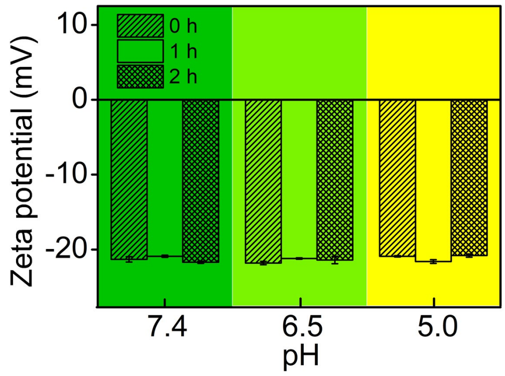

Figure S10. Zeta potentials of TEID at different pHs for different times. Zeta potentials of TEID at pH 5.0, 6.5 and 7.4 for different times.

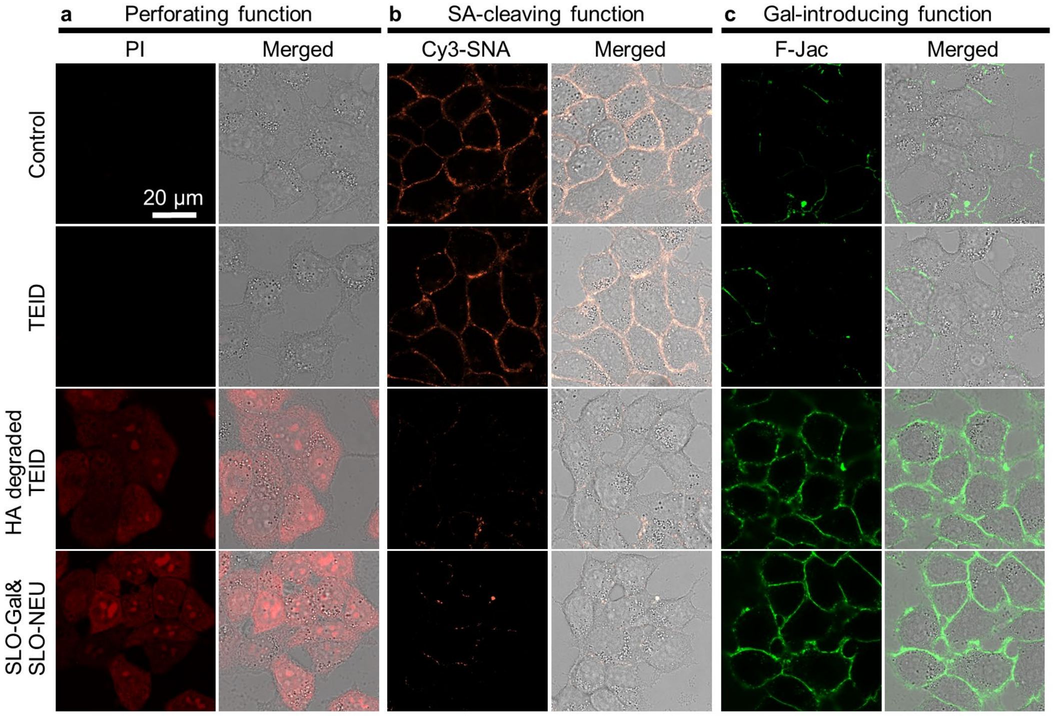

Figure S11. CLSM verification of the triple functions of TEID and the degraded TEID on MCF-7 cells. a, b) CLSM images MCF-7 cells after incubated with PBS (Control), TEID, HA degraded TEID and SLO-Gal&SLO-NEU and then stained with PI to verify perforating function (a), Cy3-SNA to verify SA-cleaving function (b). c) CLSM images of GD pre-treated MCF-7 cells after incubated with PBS (Control), TEID, HA degraded TEID and SLO-Gal&SLO-NEU and then stained with F-Jac to verify the Gal-introducing function.

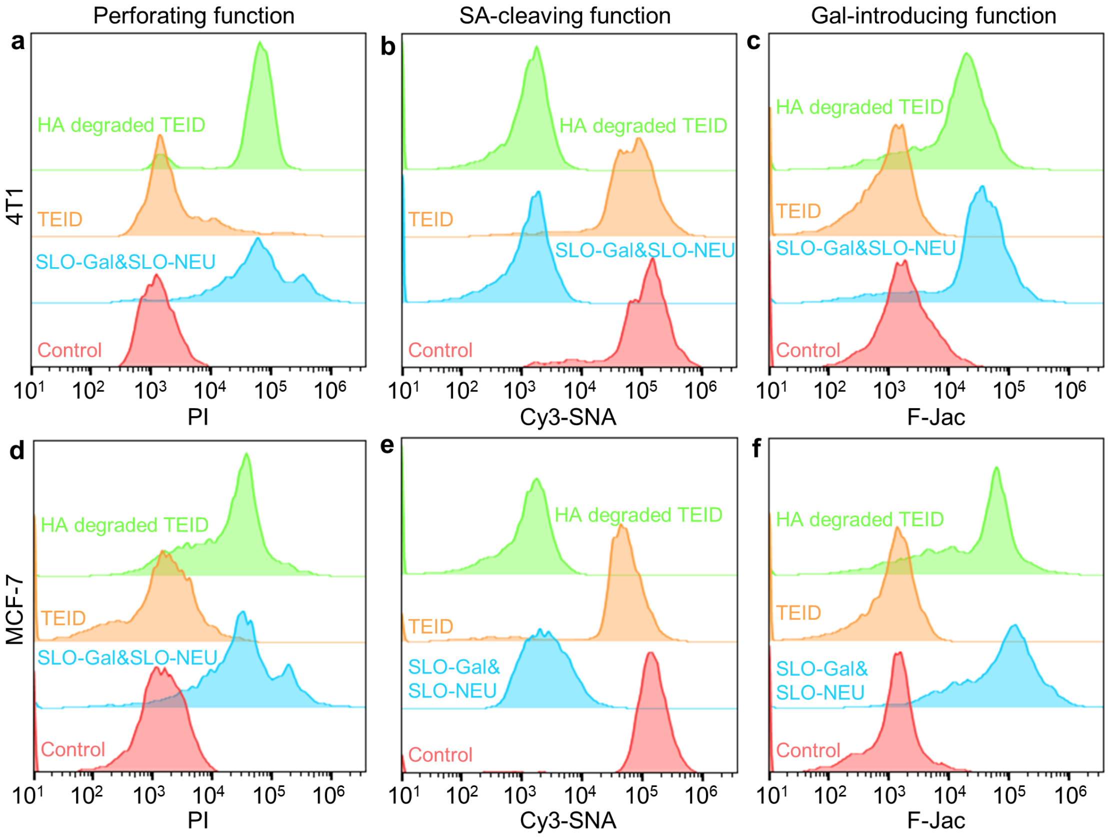

Figure S12. Flow cytometric analysis of the triple functions of TEID and the degraded TEID. a, b, d, e) Flow cytometric analysis of 4T1 or MCF-7 cells incubated with PBS (Control, red), SLO-Gal&SLO-NEU (blue), TEID (orange), HA degraded TEID (green), and then stained with PI to verify the perforating function (a, d), and Cy3-SNA to verify the SA-cleaving function (b, e). c, f) Flow cytometric analysis of GD pre-treated 4T1 or MCF-7 cells incubated with PBS (Control, red), SLO-Gal&SLO-NEU (blue), TEID (orange), HA degraded TEID (green), and then stained with F-Jac to verify the Gal-introducing function.

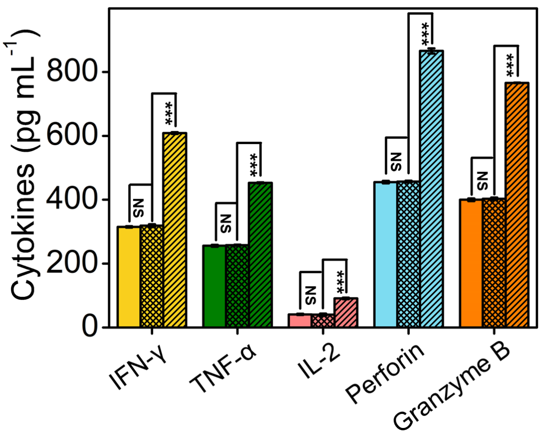

Figure S13. ELISA analysis of the secretion of cytokines from NK cells after incubation with TEID and the degraded TEID. ELISA analysis of the secreted cytokines (IFN-γ, TNF-α, IL-2, perforin and granzyme B) from NK cells after incubation with 4T1 cells pre-treated with PBS (non-shaded columns), TEID (dotted columns) and HAase degraded TEID (shaded columns) for 24 h. Statistical analysis was performed by unpaired two-tailed t-tests (***$p < 0.001$; NS, not significant), $n=3$.

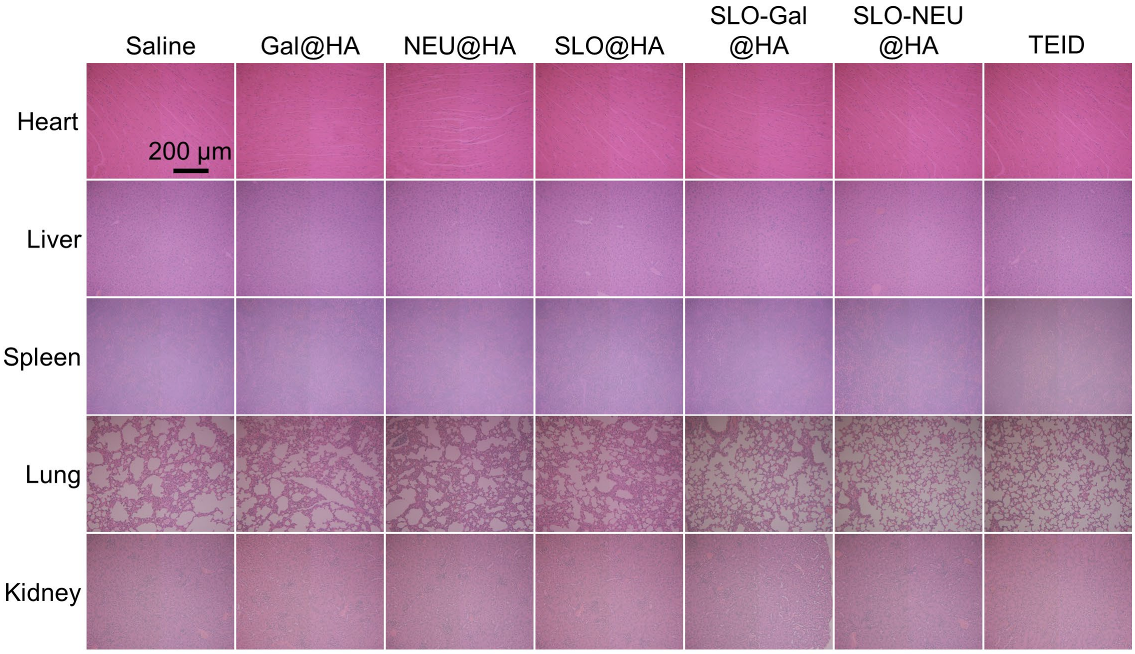

Figure S14. The side effects of TEID in the immunotherapy of tumor-bearing mice. Histological H&E staining images of heart, liver, spleen, lung and kidney collected from 4T1 tumor-bearing mice after therapy with saline, Gal@HA, NEU@HA, SLO@HA, SLO-Gal@HA, SLO-NEU@HA, and TEID at day 22.

References

[1] R. Mo, T. Y. Jiang, R. DiSanto, W. Y. Tai, Z. Gu, *Nat. Commun.* **2014**, *5*, 3364.
[2] X. F. Yu, H. F. Shi, Y. R. Li, Y. N. Guo, P. W. Zhang, G. Y. Wang, L. Li, X. Chen, L. Ding, H. X. Ju, *ACS Appl. Mater. Interfaces.* **2020**, *12*, 54387-54398.
